# Bicluster Sampled Coherence Metric (BSCM) provides an accurate environmental context for phenotype predictions

**DOI:** 10.1186/1752-0509-9-S2-S1

**Published:** 2015-04-15

**Authors:** Samuel A Danziger, David J Reiss, Alexander V Ratushny, Jennifer J Smith, Christopher L Plaisier, John D Aitchison, Nitin S Baliga

**Affiliations:** 1 Institute for Systems Biology, Seattle, WA 98109 USA; 2 Seattle Biomedical Research Institute, Seattle, WA 98109 USA

## Abstract

**Background:**

Biclustering is a popular method for identifying under which experimental conditions biological signatures are co-expressed. However, the general biclustering problem is NP-hard, offering room to focus algorithms on specific biological tasks. We hypothesize that conditional co-regulation of genes is a key factor in determining cell phenotype and that accurately segregating conditions in biclusters will improve such predictions. Thus, we developed a bicluster sampled coherence metric (BSCM) for determining which conditions and signals should be included in a bicluster.

**Results:**

Our BSCM calculates condition and cluster size specific p-values, and we incorporated these into the popular integrated biclustering algorithm cMonkey. We demonstrate that incorporation of our new algorithm significantly improves bicluster co-regulation scores (p-value = 0.009) and GO annotation scores (p-value = 0.004). Additionally, we used a bicluster based signal to predict whether a given experimental condition will result in yeast peroxisome induction. Using the new algorithm, the classifier accuracy improves from 41.9% to 76.1% correct.

**Conclusions:**

We demonstrate that the proposed BSCM helps determine which signals ought to be co-clustered, resulting in more accurately assigned bicluster membership. Furthermore, we show that BSCM can be extended to more accurately detect under which experimental conditions the genes are co-clustered. Features derived from this more accurate analysis of conditional regulation results in a dramatic improvement in the ability to predict a cellular phenotype in yeast. The latest cMonkey is available for download at https://github.com/baliga-lab/cmonkey2. The experimental data and source code featured in this paper is available http://AitchisonLab.com/BSCM. BSCM has been incorporated in the official cMonkey release.

## Background

Biclustering is a technique for examining mRNA expression data and discovering genes that are conditionally co-regulated -i.e., genes that have common expression patterns under certain conditions, but not under others [1]. Thus biclustering is a valuable tool for analysing large gene expression datasets, particularly when those data have been generated under multiple experimental conditions. As mRNA expression data have become ever more plentiful, many diverse public datasets have become available. While it remains difficult to make the most biological sense of this largess, biclustering has been successfully used to mine it for novel biological relationships, to correlate environmental condition with expression patterns, and to predict gene expression under new conditions not in the original datasets [2].

cMonkey is a particularly powerful biclustering tool that finds putatively co-regulated genes by combining mRNA expression levels (or similar measurements), *de novo *detected TF binding motifs, and networks of known gene associations [3]. It was originally developed to reconstruct regulatory networks for *Halobacterium salinarum *[4]. Since then, cMonkey has been continuously developed and has been applied to discover novel biology in other organisms such as humans [5] and *Saccharomyces cerevisiae (S. cerevisiae) *[2], revealing novel challenges. One challenge is building biclusters on consortium datasets containing expression data generated in multiple labs using different mRNA measurement technologies and yeast grown under drastically different conditions. These compendium experiments potentially have different noise levels and can be difficult to compare.

While cMonkey is an effective tool for these circumstances, we found that the mRNA expression evaluation model used by existing versions of cMonkey does not handle such situations as well as it could. It quantifies bicluster coherence by comparing the measured distribution for each gene in a bicluster to an idealized normal distribution, which is based upon the mean expression of the other genes in the bicluster, and the expected variance for each experiment with a uniform systematic error constant. This uniform variance assumption is often inaccurate for expression compendia, because multiple measurement technologies applied in multiple labs will almost certainly have different errors associated with them.

Biclustering of gene expression measurements continues to be an active area of research, and there has been significant progress in improving gene expression biclustering [6], however very little of it has focused on combining multiple datasets from disparate sources, such as are available from GEO (the gene expression omnibus) [7,8]. Classical gene expression biclustering, based upon co-expression heuristics such as the Cheng and Church mean-squared-residue [9], have achieved impressive methodological diversity and results [10]. However, the original cMonkey implementation instead used a probabilistic model that enabled a more rigorous integration of co-expression with bicluster evidence based on non-gene expression data types [3]. Other methods have focused on biclustering in the context of specific biological problems. Reference gene biclustering finds biclusters that match the expression pattern for a single reference gene [11]. Differential co-expression biclustering finds biclusters that are differentially co-expressed between two conditions [12]. Time series biclustering finds genes that follow common temporal co-expression patterns as revealed in time series data [13]. However, none of these methods is well suited to analyse variable compendium data and discover globally relevant biclusters. Reference gene biclustering will only find biclusters relevant for a single reference gene; differential co-expression biclustering requires exactly two well annotated datasets; and time series biclustering requires time series data. As variable compendium data can contextualize behaviour and reveal novel biology that a single condition specific dataset cannot [2], it is important to develop a metric appropriate for analysing these diverse data sets.

**Figure 1:**
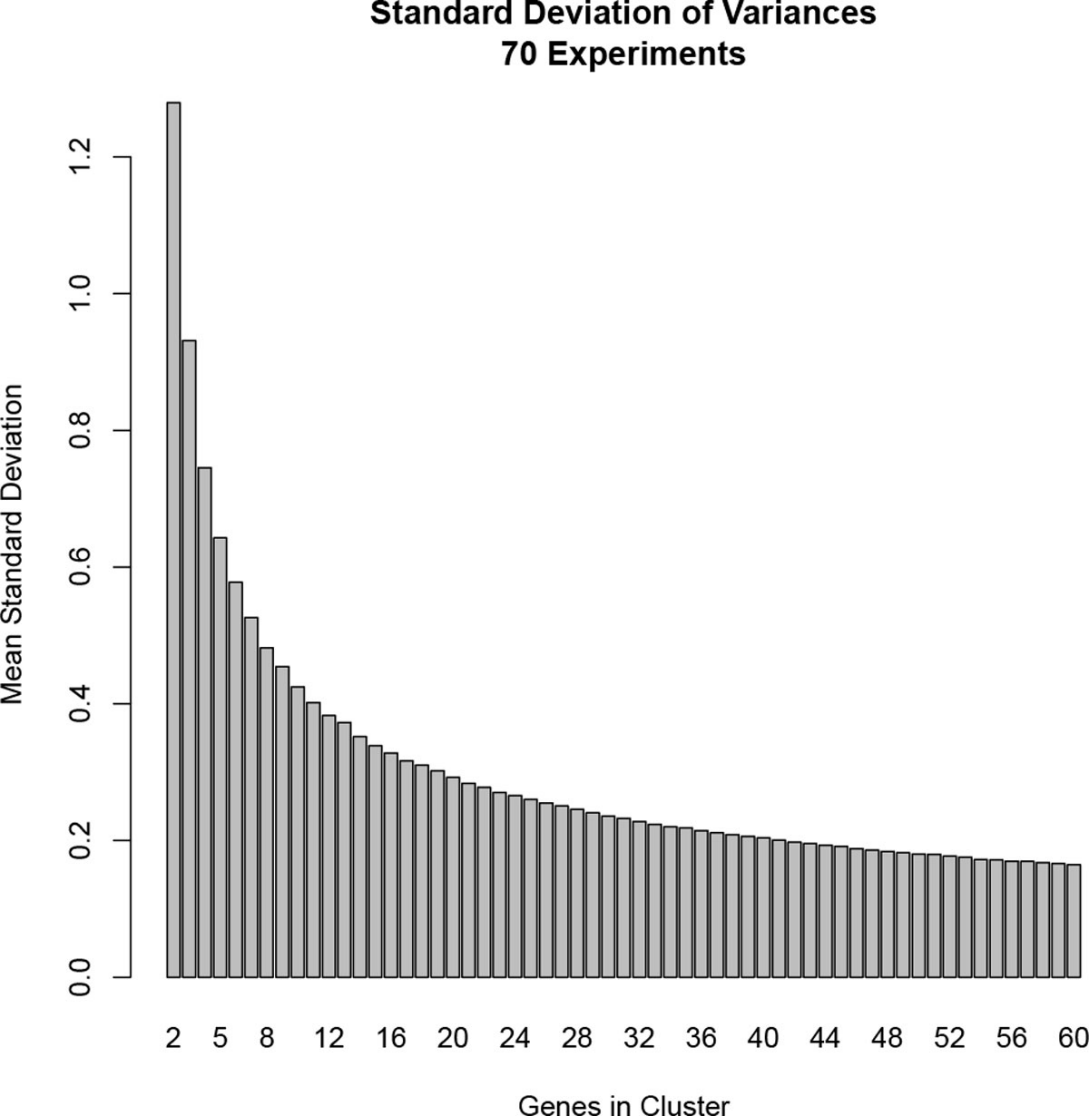
**Background Cluster Variance Decreases with Cluster Size**. Shown is the standard deviation of gene variances when genes are sampled from a 70 experiment ***S. cerevisiae ***dataset.

Therefore, we developed our bicluster sampled coherent metric (BSCM). BSCM calculations modify the original cMonkey co-expression model, in order to treat the genome-wide measurements from individual experiments independently. Specifically, a new background distribution is calculated empirically for each experiment and each cluster size. This removes the uniform systematic error term and, as shown in Figure 1, accounts for the effects of cluster size on expected coherence (thus removing the need for a user-defined prior distribution).

**Figure 2:**
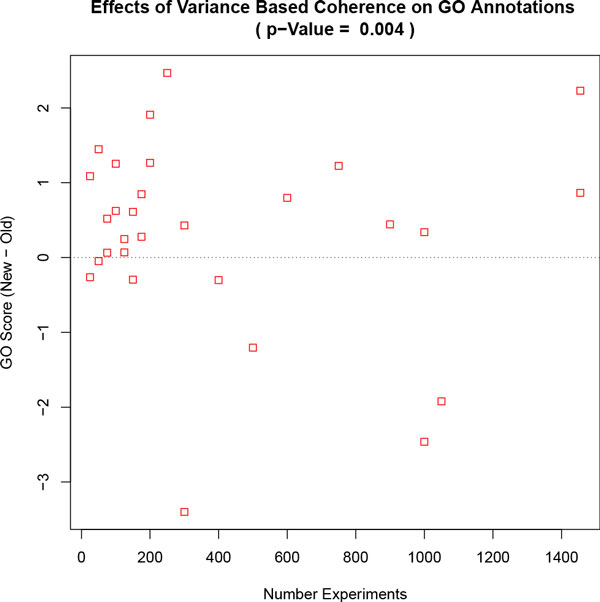
**Improved GO Co-expression with Larger Dataset**. Shown are the changes in the GOScores comparing clusters built with BSCM versus the previous method. The horizontal line indicates what the average score would be if the new and old methods were equally good. The p-value is calculated using a two-tailed paired t-test between the BSCM and non-BSCM GOScores.

Running cMonkey with this refined BSCM improves the conditional co-regulation of genes assigned to each bicluster. cMonkey has an internal scoring function that (without using BSCM) estimates bi-cluster quality by considering gene co-expression, known protein and genetic interactions, and the quality of common upstream binding motifs [3]. Using a test dataset consisting of 252 *Mycoplasma pneumonia (M. pneumoniae) *experiments [6], the new coherence metric improved the score in 75 out of 125 runs (binomial p-value < = 0.01). We then applied a similar test to *S. cerevisiae *(Additional File 1, [1]), but measured potentially more biological relevant Gene Ontology (GO) annotation [7] enrichments to score the clusters and found improvement in 21 of 29 experiments using the new co-expression p-value (Figure 2, binomial p-value = 0.004).

**Figure 3:**
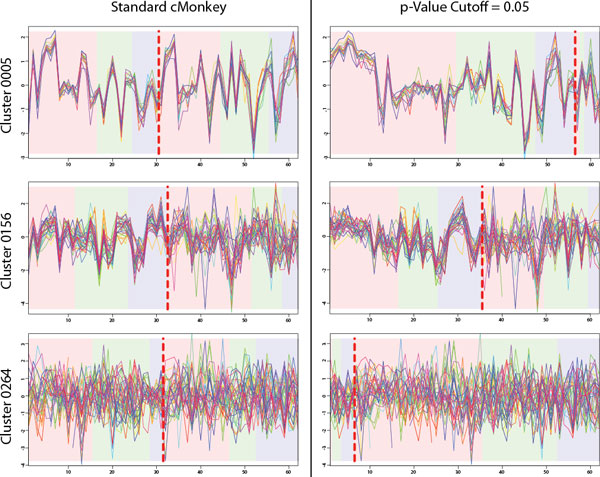
**Re-splitting Biclusters Based on BSCM**. These biclusters were built on *S. cerevisiae *grown in batch culture and chemo stat and run through a microarray at different time points. Those conditions to the left of the dotted red line are included in the cluster and those to the right are excluded. The variance based p-value is applied to re-split the biclusters and change which conditions are included. Cluster 0005 contains 15 ribosomal genes that are expected to be co-regulated. Re-splitting increases the number of included conditions from 30/62 to 56/62. Cluster 0156 contains 31 genes related to mitosis, DNA damage, and metabolism. Re-splitting increases the number of included conditions from 32/62 to 35/62. Cluster 0229 contains 34 genes of mostly unknown function although some were related to carbohydrate metabolism. Re-splitting reduces the number of included conditions from 34/62 to 18/62.

Another important aspect of biclustering and cMonkey is to select under which experimental conditions genes in a bicluster are co-expressed (i.e. conditional co-expression). Existing versions of cMonkey do this using a method that classifies half of all experimental conditions (on average) as part of each cluster. This method is limited because genes under certain experimental conditions would be considered not co-expressed simply because they were slightly more coherently expressed under other experimental conditions and vice versa (Figure 3). BSCM provides a more robust method to determine which experiments belong in a bicluster: with a p-value cutoff of 0.05.

**Figure 4:**
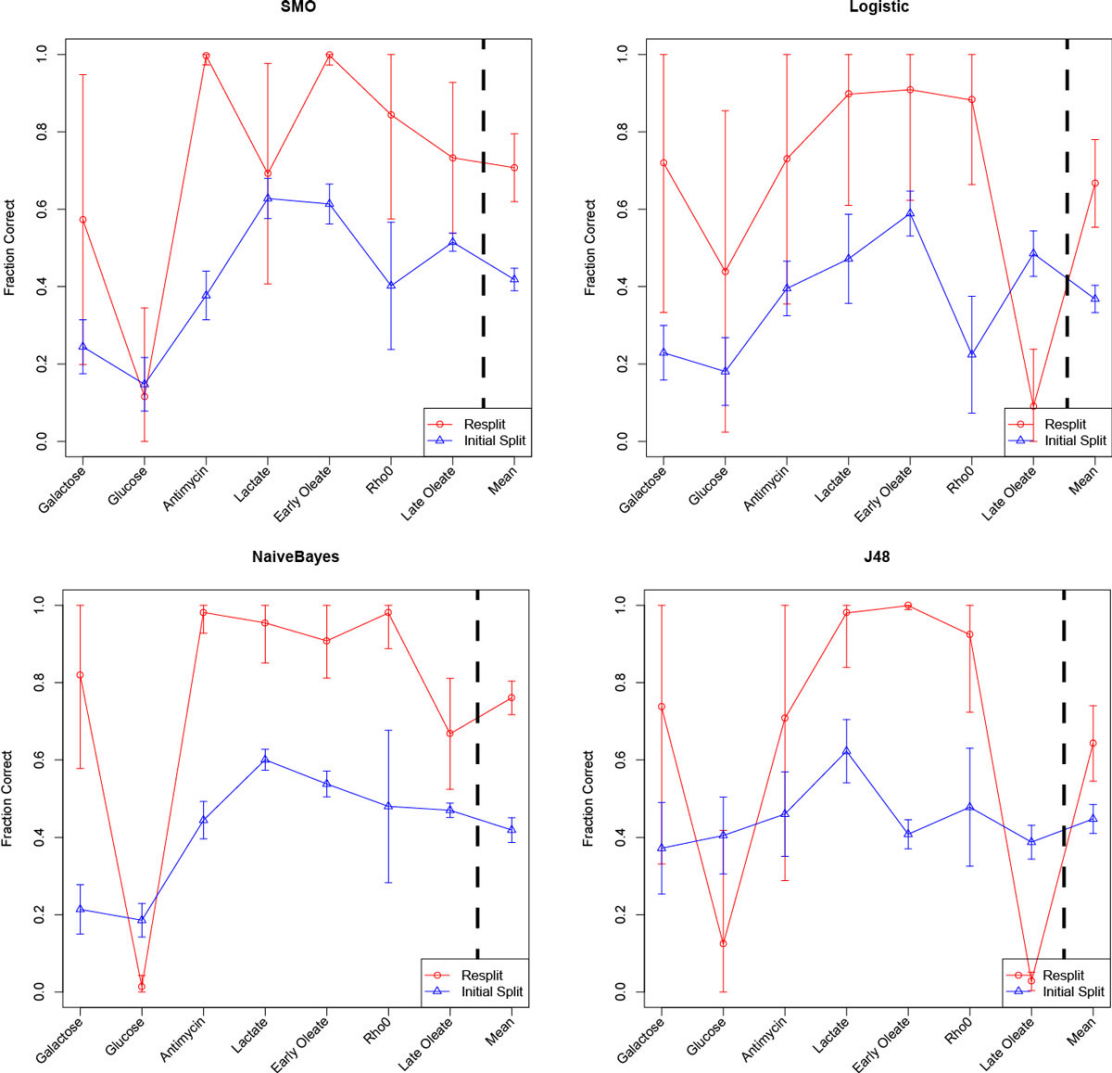
**Peroxisome Proliferation Classification with Re-split Clusters**. Predictions were made using the Naïve Bayes, Support Vector Machine (SMO), Logistic Regression, and J48 decision tree classifiers. Error bars show one standard deviation. Mean refers to the mean fraction correct for the seven experimental conditions. Two-tailed paired t-test p-values are less than 10**^-23 ^**for all experimental conditions (n = 100).

To test if this BSCM indeed improves cMonkey's ability to accurately detect condition dependant bicluster coherence, we tested the quality of the biclusters with a biological application. We used compendium data to predict which growth conditions induce peroxisomes to proliferate[2,8-14]. Peroxisomes are organelles that perform a variety of functions including the metabolism of fatty acids. In yeast, peroxisomes are conditionally required, and their size and abundance can change dramatically with growth condition. Peroxisomes proliferation is: 1) repressed by fermentative growth-conditions such as glucose and galactose [15]; 2) de-repressed under non-fermentative growth such as glycerol, lactate, pyruvate, oxylacetate, acetate, fatty acids (e.g. oleate), antimycin, and the lack of mitochondrial DNA [16-18]. Peroxisome proliferation is controlled at the level of transcription by up-regulation genes involved in peroxisome biogenesis and function [15]. To predict conditions of peroxisome proliferation using biclusters, we used conditional co-expression features to build a classifier to predict conditional dynamics of peroxisome proliferation. We compared the existing cMonkey biclusters to BSCM resplit biclusters and found that this greatly improved cross-validated predictions of peroxisome proliferation (Figure 4).

## Results & discussion

In cMonkey, the coherence p-value for a gene *i *in cluster *k *is referred to as *r_ik_*. Mathematically, cMonkey improves the coherence of its biclusters by minimizing *r_ik _*for all genes in each cluster (subject to other constraints). BSCM changes how *r_ik _*is calculated. By thus improving the co-expression p-value function with BSCM, we were able to improve the overall quality of the biclusters. We assess this improvement using three metrics: 1) We use cMonkey's internal scoring which calculated overall cluster quality using the non-BSCM *r_ik _*and test on *M. pneumoniae*; 2) We use a GO term enrichment score and test on *S. cerevisiae*; and 3) We use the experiments included in clusters to build a classifier that predicts peroxisome proliferation in *S. cerevisiae*.

### Bicluster Sampled Coherence Metric (BSCM) improves *M. pneumoniae *model

We compared cMonkey biclusters derived using our updated BSCM-based p-value with those of the previous version (i.e. version 4.8.2). We ran each version 125 times on the small, quickly calculated, *M. pneumoniae *dataset [6]. The average score (Equation 2) for each bicluster was improved in 75 out of 125 runs when we used our BSCM co-expression p-value (binomial p-value = 0.009), and also showed similar improvement across other metrics (Table 1). Importantly, because we used the Equation 2 scoring function, the coherence portion of the score was calculated using the old coherence p-value (*r_ik_*). Thus the new scores were better, even when the evaluation was biased towards the non-BSCM *r_ik_*.

**Table 1:** BiCluster Quality Score on *M. pneumoniae (MPN)*

	Non-BSCM	BSCM	Significance p-value
**n**	125	125	

**Score**	-12.26	-12.34	2.40E-003

**Improved Score**	75/125	50/125	9.84E-003

**Mean p-value**	0.129	0.109	2.20E-016

**cMonkey Version**	4.8.2	4.8.2	

Non-BSCM refers to cMonkey runs that used the classical cMonkey cluster coherence score. BSCM refers to the new coherence p-value discussed in this paper. n refers to the number of cMonkey runs. Score refers to the average cluster score used to determine bicluster quality in cMonkey (lower is better). Improved Score refers to the number of cMonkey runs in which a coherence scoring method has a lower Score. Mean p-value refers to the average p-value for all experiments in all clusters for all cMonkey runs. Significance p-value compares the old to the new using a t- test for Score, binomial distribution for Improved Score, and t-test for Mean p-value.

### Bicluster Sampled Coherence Metric (BSCM) improves *S. cerevisiae *model

We further tested BSCM using a *S. cerevisiae *dataset consisting of 26 public sets resulting in 1455 experiments [8-10,19-41] (Additional File 1). *S. cerevisiae *has over 6,000 genes compared to 688 for *M. pneumoniae *so it was impractical to run cMonkey 125 times for the entire *S. cerevisiae *dataset. However, because *S. cerevisiae *is much better annotated, it was possible to use a GO annotation enrichment based scoring metric (GOScore, Equation 5) that was independent of cMonkey's scoring function. We identified 29 random experiment subsets with 50-1445 microarrays each, eliminated genes without large expression changes, and then ran cMonkey with both the BSCM and non-BSCM based p-values. We applied the GOScore and found improvement in 21 of 29 experiments using the new BSCM p-value (Figure 2, binomial p-value = 0.004).

### New BSCM allows more accurate bicluster inclusion

The primary advantage of biclustering over standard clustering is that biclusters include the notion of conditional inclusion. That is to say that the genes in the bicluster are conditionally co-expressed under certain experimental conditions, but not under others. The original cMonkey implementation assumed (via a prior probability) that approximately half of all experiments included in a cluster should be included, and half should be excluded. However, as shown in the left panel of Figure 3, this did not work well in conditions where the genes are co-regulated under all conditions (such as was the case for ribosomal biclusters), or in clusters where the genes are co-regulated under a very small subset of conditions. By contrast, the new BSCM provided a natural cutoff for re-splitting biclusters. As shown in Equation 3, *r_ik _*estimates the p-value for each experiment *j *, given a cluster *k*. Those experiments where *r_ik _*≤ 0.05 are included in the cluster, all others are excluded.

These new splits were more visually satisfying (Figure 3, right panel), however we were interested in determining if the re-split clusters were biologically more relevant. To test this we built a classifier that would predict if yeast would proliferate peroxisomes under certain conditions based on whether or not experiments performed under those conditions were included or excluded from biclusters. We assembled a dataset of relevant conditions (see Methods), extracted the features, and tried four common machine learning algorithms (Figure 4). The classifier performed similarly well regardless of the machine learning algorithm, but the patterns were most obvious when using a Naïve Bayes classifier. Using this classifier, overall peroxisome proliferation prediction accuracy improves from 41.9% to 76.1% correct when using the BSCM bicluster inclusion rather than the previous method. The classifier accuracy was nearly perfect (>95%) for four of the seven conditions, while it is poor only for predictions of glucose. This probably reflects a biological reality: the glucose response pathway is included in the galactose response, but not vice versa. Thus, the information necessary for understanding the galactose response is present when glucose is in the training set. However, when only galactose is present in the training set, a key piece of information is missing necessary to inform the classifier.

## Conclusions

mRNA expression data is becoming ever more plentiful as microarrays become more commonplace or are replaced by multiplexed RNA-seq technology. The improved Bicluster Sampled Coherence Metric (BSCM) provides a better way to simplify and interpret large amounts of expression data that come from multiple sources. Beyond directly improving biclusters, this algorithm is useful for drawing additional information out of each bicluster and using it to train a classifier. We anticipate that this method will become particularly relevant for the broad bioinformatics community interested in humans -- where each cell type may be regarded in the same manner as yeast or bacteria in different environmental conditions. This opens the potential to classify cell types based on mRNA signatures, and to reveal conditions or perturbations that induce a specific cellular response.

## Methods

Let *I *represent the set of all genes, *J *all experiments, and *K *all biclusters. A bicluster k∈K contains genes *I_k_*, where each gene is i∈I, and includes experiments $j\in J_{k}$ such that $J_{k}\subseteq J$.

In the original cMonkey [3], the variance for each experiment *j *is calculated as $\sigma_{j}^{2}=|I{|}^{-1}\sum_{i\in I}{\left(x_{ij}-{\bar{x}}_{j}\right)}^{2}$ where *x_ij _*is the expression level for gene *i *in experiment *j *and ${\bar{x}}_{j}=\sum_{i\in I}x_{ij}/|I|$. The likelihood for a given *x_ij _*in cluster *k *is

(1) $$p\left(x_{ij}\right)=\frac{1}{\sqrt{2\pi \left(\sigma_{j}^{2}+\varepsilon^{2}\right)}}\text{exp}\left[-\frac{{\left(x_{ij}-{\bar{x}}_{jk}\right)}^{2}+\varepsilon^{2}}{\sigma_{j}^{2}+\varepsilon^{2}}\right]$$

where ε is a constant error term, ${\bar{x}}_{jk}^{}=\sum_{i\in I}x_{ij}/|I_{k}|$, and $I_{k}$ is the genes in cluster *k*. The co-expression p-value, *r_ik_*, for each gene *i *is derived from Equation (1). This is combined with weighted log p-values calculated for the TF binding motifs (*Q_ik_*) and known gene associations (*S_ik_*) as $g_{ik}=r_{o}\text{log}{\tilde{r}}_{ik}+Q_{ik}+S_{ik}$ where $\text{log}{\tilde{r}}_{ik}$ is the a z-score normalized version of $\text{log}r_{ik}$ and *r_o _*is a weight for adjusting the relative importance of *r_ik_*. A final score for each bicluster is calculated as

(2) $$\text{scor}{\text{e}}_{k}=\underset{i\in I_{k}}{\sum }g_{ik}/|I_{k}|$$

### Bicluster Sampled Coherence Metric (BSCM) method

Here we change how the co-expression p-value, *r_ik _*was calculated as follows:

(3) $$r_{jk}=\frac{1}{\sqrt{2\pi \sigma_{{\bar{\sigma }}_{j|k|}}^{2}}}\text{exp}\left[-\frac{\sigma_{jk}^{2}-{\bar{\sigma }}_{j|k|}^{2}}{\sigma_{{\bar{\sigma }}_{j|k|}}^{2}}\right]$$

(4) $$r_{ik}=\underset{j\in J_{k}}{\sum }\frac{r_{jk}}{\left|J_{k}\right|}$$

${\bar{\sigma }}_{j|k|}$ is the mean variance for the number of genes in bicluster *k *as determined bootstrap sampling. $\sigma_{{\bar{\sigma }}_{j|k|}}^{2}$ is the standard deviation of the values used to calculate ${\bar{\sigma }}_{j|k|}$. The background distribution is calculated for each condition j∈J and for each number of genes that occurs in a given bicluster *k *by sampling |*k*| genes 200 times from experimental condition *j *and drawing additional samples in sets of 200 until ${\bar{\sigma }}_{j|k|}$ and $\sigma_{{\bar{\sigma }}_{j|k|}}^{2}$ change by less than 1%. To determine which genes should be added or removed from a cluster, we calculate a new *r_ik _*supposing gene *i *were added or removed. As a practical matter, background distributions for are pre-calculated for all cluster sizes less than or equal to the maximum size represented in the initial seed clusters, and additional background distributions are calculated as needed during program execution.

### Cluster scoring based on GO terms

To independently evaluate the quality of the clusters, we calculate a Gene Ontology[7] based GOScore from the binomial enrichment of GO slim terms, *G*.

(5) $$GOScore=\underset{k}{\sum }\overset{G}{\underset{g}{\sum }}-\text{log}\left(\text{pG}{\text{O}}_{k,g}\right)$$

where $\text{pG}{\text{O}}_{k,g}$ is the enrichment p-value for term *g *in cluster *k*.

### Classifier construction

We tested whether *r_ik _*could be used with a p-value cutoff of 0.05 to predict if experimental conditions would result in peroxisome proliferation ("YES") or not ("NO"). We built 544 yeast biclusters using 233 experiments in seven different experimental conditions with known peroxisome proliferation: thirty glucose ("NO"), twenty early oleate ("YES"), and twenty-one late oleate experiments ("YES")[2], seventy-five galactose ("NO"), eighteen lactate ("YES"), five rho- ("YES"), and sixty-four antimycin ("YES") experiments [8,9,13,17]. For every bicluster, each of the 233 experiments was assigned a value indicating whether genes are "UP" or "DOWN" -regulated if included in a given bicluster, or "EXCLUDED" otherwise. Many experiments were replicates, so standard n-fold cross-validation was inappropriate. Therefore, each of the seven growth-conditions was treated as a splitting boundary. Thus when the classifier predicted proliferation in antimycin, antimycin was absent from the training set. During each split we downsampled, thus providing stochastisticity. Predictions were made using decision trees, logistic regression, support vector machines (SVMs), and naive bayes [42,43]. (See supplemental code and data for implementation.)

## Additional information

This file contains code and data necessary to run the experiments presented in this paper. Available at http://AitchisonLab.com/BSCM/TestData.BSCM.tar.gz (156 MB)

## Competing interests

The authors declare that they have no competing interests.

## Authors' contributions

SAD designed and implemented the Bicluster Sampled Coherence Metric (BSCM) as well as the experiments, DJR provided necessary consulting as to the inner workings for cMonkey, AVR designed several of the analysis techniques, CLP suggested the basic form of the new BSCM based p-value metric, JJS consulted on peroxisome biology and validated datasets, and JDA & NSB oversaw all aspects of algorithm development and experimentation. All authors have drafted and revised the manuscript and approved the final version.

## Supplementary Material

Additional File 1 **Contains details about the public datasets download from GEO**. Click here for file

## Declarations

Publication of this article has been funded by grants from the National Institutes of Health (P41GM109824, P50 GM076547, R01 GM075152, 1R01 GM077398, and U54GM103511) and the National Science Foundation (DBI-0640950).

This article has been published as part of *BMC Systems Biology *Volume 9 Supplement 2, 2015: Selected articles from the IX International Conference on the Bioinformatics of Genome Regulation and Structure\Systems Biology (BGRS\SB-2014): Systems Biology. The full contents of the supplement are available online at http://www.biomedcentral.com/bmcsystbiol/supplements/9/S2.
