# Supplementary material for: Bicluster Sampled Coherence Metric (BSCM) provides an accurate environmental context for phenotype predictions
Source: BMC Syst Biol. 2015 Apr 15;9(Suppl 2):S1. doi: 10.1186/1752-0509-9-S2-S1 (PMC4407105; doi:10.1186/1752-0509-9-S2-S1)
Supplement: Additional File 1 — Contains details about the public datasets download from GEO. [file 1752-0509-9-S2-S1-S1.PDF]

Supplementary Table 1: Yeast Data Taken from Gene Expression Omnibus (Edgar *et al.*, 2002)

Experiment Type	*GEO Accession*	*Reference*
**Histone H2B**	GDS2029	(Parra *et al.*, 2006; Nag *et al.*, 2010)
**Kinome**	GSE25644	(van Wageningen *et al.*, 2010)
**Organic Acid**	GDS2925	(Abbott *et al.*, 2007)
**UPF1**	GDS1611	(Guan *et al.*, 2006)
**Ribosomes**	GDS3061	(Komili *et al.*, 2007)
**Dehydration**	GDS2715	(Singh *et al.*, 2005)
**Wine**	GDS3332	(Marks *et al.*, 2008)
**Limited Media**	GDS777	(Knijnenburg *et al.*, 2007; Tai *et al.*, 2005; Boer *et al.*, 2003)
**Antimycin**	GSE3705	(Lai *et al.*, 2008)
**Metabolic**	GDS2267	(Tu *et al.*, 2005)
**Spores in Glucose**	GSE7362	(Joseph-Strauss *et al.*, 2007)
**Anaerobic**	GDS2003	(Lai *et al.*, 2005)
**Splicing Mutants**	GDS759	(Sapra *et al.*, 2004)
**Microgravity**	GDS1687	(Sheehan *et al.*, 2007)
**Carbon Source**	GDS1752	(Ronen and Botstein, 2006)
**Alpha**	GSE8982	None Reported
**Oxidative**	GDS3035	(Komili *et al.*, 2007)
**Cytotoxic**	GDS1299	(Caba *et al.*, 2005, caba)
**Caffeine**	GDS2914	(Kuranda *et al.*, 2006)
**Rho^0**	GSE15302	(Veatch *et al.*, 2009)
**Sporulation**	GSE8506	None reported.
**Gentamicin**	GDS2999	(Komili *et al.*, 2007)
**Pterostilbene**	GDS3245	(Pan *et al.*, 2008)
**Caffeine TOR1**	GDS2336	(Reinke *et al.*, 2006)
**Pyocyanin**	GDS2522	(Angell *et al.*, 2006)
**Filamentous**	GDS608	(Prinz *et al.*, 2004)
**Oleate**	N/A	Not yet submitted to Gene Expression Omnibus

Abbott,D.A. *et al.* (2007) Generic and specific transcriptional responses to different weak organic acids in anaerobic chemostat cultures of Saccharomyces cerevisiae. *FEMS Yeast Res.*, **7**, 819–833.
Angell,S. *et al.* (2006) Pyocyanin isolated from a marine microbial population: synergistic production between two distinct bacterial species and mode of action. *Chem. Biol.*, **13**, 1349–1359.
Boer,V.M. *et al.* (2003) The genome-wide transcriptional responses of Saccharomyces cerevisiae grown on glucose in aerobic chemostat cultures limited for carbon, nitrogen, phosphorus, or sulfur. *J. Biol. Chem.*, **278**, 3265–3274.
Caba,E. *et al.* (2005) Differentiating mechanisms of toxicity using global gene expression analysis in Saccharomyces cerevisiae. *Mutat. Res.*, **575**, 34–46.
Edgar,R. *et al.* (2002) Gene Expression Omnibus: NCBI gene expression and hybridization array data repository. *Nucleic Acids Research*, **30**, 207 –210.
Guan,Q. *et al.* (2006) Impact of nonsense-mediated mRNA decay on the global expression profile of budding yeast. *PLoS Genet.*, **2**, e203.
Joseph-Strauss,D. *et al.* (2007) Spore germination in Saccharomyces cerevisiae: global gene expression patterns and cell cycle landmarks. *Genome Biol.*, **8**, R241.
Knijnenburg,T.A. *et al.* (2007) Exploiting combinatorial cultivation conditions to infer transcriptional regulation. *BMC Genomics*, **8**, 25.
Komili,S. *et al.* (2007) Functional specificity among ribosomal proteins regulates gene expression. *Cell*, **131**, 557–571.
Kuranda,K. *et al.* (2006) Investigating the caffeine effects in the yeast Saccharomyces cerevisiae brings new insights into the connection between TOR, PKC and Ras/cAMP signalling pathways. *Mol. Microbiol.*, **61**, 1147–1166.
Lai,L.-C. *et al.* (2008) Comparison of the transcriptomic ‘stress response’ evoked by antimycin A and oxygen deprivation in Saccharomyces cerevisiae. *BMC Genomics*, **9**, 627.
Lai,L.-C. *et al.* (2005) Dynamical remodeling of the transcriptome during short-term anaerobiosis in Saccharomyces cerevisiae: differential response and role of Msn2 and/or Msn4 and other factors in galactose and glucose media. *Mol. Cell. Biol.*, **25**, 4075–4091.
Marks,V.D. *et al.* (2008) Dynamics of the yeast transcriptome during wine fermentation reveals a novel fermentation stress response. *FEMS Yeast Res.*, **8**, 35–52.
Nag,R. *et al.* (2010) A cassette of N-terminal amino acids of histone H2B are required for efficient cell survival, DNA repair and Swi/Snf binding in UV irradiated yeast. *Nucleic Acids Res.*, **38**, 1450–1460.
Pan,Z. *et al.* (2008) Identification of molecular pathways affected by pterostilbene, a natural dimethylether analog of resveratrol. *BMC Med Genomics*, **1**, 7.
Parra,M.A. *et al.* (2006) Deciphering the roles of the histone H2B N-terminal domain in genome-wide transcription. *Mol. Cell. Biol.*, **26**, 3842–3852.
Prinz,S. *et al.* (2004) Control of yeast filamentous-form growth by modules in an integrated molecular network. *Genome Res.*, **14**, 380–390.
Reinke,A. *et al.* (2006) Caffeine targets TOR complex I and provides evidence for a regulatory link between the FRB and kinase domains of Tor1p. *J. Biol. Chem.*, **281**, 31616–31626.
Ronen,M. and Botstein,D. (2006) Transcriptional response of steady-state yeast cultures to transient perturbations in carbon source. *Proc. Natl. Acad. Sci. U.S.A.*, **103**, 389–394.
Sapra,A.K. *et al.* (2004) Genome-wide analysis of pre-mRNA splicing: intron features govern the requirement for the second-step factor, Prp17 in Saccharomyces cerevisiae and Schizosaccharomyces pombe. *J. Biol. Chem.*, **279**, 52437–52446.
Sheehan,K.B. *et al.* (2007) Yeast genomic expression patterns in response to low-shear modeled microgravity. *BMC Genomics*, **8**, 3.
Singh,J. *et al.* (2005) Transcriptional response of Saccharomyces cerevisiae to desiccation and rehydration. *Appl. Environ. Microbiol.*, **71**, 8752–8763.

Tai,S.L. *et al.* (2005) Two-dimensional transcriptome analysis in chemostat cultures. Combinatorial effects of oxygen availability and macronutrient limitation in Saccharomyces cerevisiae. *J. Biol. Chem.*, **280**, 437–447.
Tu,B.P. *et al.* (2005) Logic of the yeast metabolic cycle: temporal compartmentalization of cellular processes. *Science*, **310**, 1152–1158.
Veatch,J.R. *et al.* (2009) Mitochondrial dysfunction leads to nuclear genome instability via an iron-sulfur cluster defect. *Cell*, **137**, 1247–1258.
van Wageningen,S. *et al.* (2010) Functional overlap and regulatory links shape genetic interactions between signaling pathways. *Cell*, **143**, 991–1004.
